# Supplementary material for: Use of Drug Claims Data and a Medication Risk Score to Assess the Impact of CYP2D6 Drug Interactions among Opioid Users on Healthcare Costs
Source: J Pers Med. 2021 Nov 10;11(11):1174. doi: 10.3390/jpm11111174 (PMC8622634; doi:10.3390/jpm11111174)
Supplement: Supplementary file 1 [file jpm-11-01174-s001.zip › jpm-1399748-supplementary.pdf]

Supplementary Materials

Table S1. Detailed CYP2D6 co-medications among CYP2D6 opioid users.

**CYP2D6 Opioid and CYP2D6 co-medications (*n* = 577)**	*n*	**Frequency %**
**CYP2D6 Inhibitors**	13	2.3%
ORPHENADRINE	6	1%
TERBINAFINE	4	0.7%
AMIODARONE	3	0.5%
**CYP2D6 substrate-MBI ***	39	6.7%
PAROXETINE	36	6.2%
MIRABEGRON	3	0.5%
**CYP2D6 substrates with Strong or Intermediate affinity**	454	78.7%
BUPROPION	108	18.7%
FLUOXETINE	89	15.4%
ARIPIPRAZOLE	13	2.3%
FLECAINIDE	2	0.3%
FLUVOXAMINE	1	0.2%
DULOXETINE	124	21.5%
CARVEDILOL	36	6.2%
PROPRANOLOL	25	4.3%
ESCITALOPRAM	15	2.6%
METOCLOPRAMIDE	12	2.1%
CITALOPRAM	9	1.6%
HYDROXYZINE	9	1.6%
DOXEPIN	4	0.7%
RISPERIDONE	3	0.5%
HALOPERIDOL	2	0.3%
LORATADINE	1	0.2%
TAMOXIFEN	1	0.2%

*MBI; mechanism-based inhibitor.

Table S2. The top 25 prescribed medications in the overall study population.

No-Opioid group		Opioid group*	
Medication Name	Number of patients (%)	Medication Name	Number of patients (%)
ETHINYLESTRADIOL	5217 (11.2%)	ACETAMINOPHEN	2449 (59.9%)
LISINOPRIL	4383 (9.4%)	AMOXICILLIN	364 (8.9%)
HYDROCHLOROTHIAZIDE	4381 (9.4%)	HYDROCHLOROTHIAZIDE	360 (8.8%)
ATORVASTATIN	3917 (8.4%)	LISINOPRIL	349 (8.5%)
THYROXINE	3753 (8.0%)	GABAPENTIN	342 (8.4%)
AMOXICILLIN	3145 (6.7%)	ATORVASTATIN	305 (7.5%)
METFORMIN	2562 (5.5%)	GUAIFENESIN	304 (7.4%)
OMEPRAZOLE	2341 (5.0%)	IBUPROFEN	291 (7.1%)
ALBUTEROL	2192 (4.7%)	OMEPRAZOLE	259 (6.3%)
LOSARTAN	2109 (4.5%)	THYROXINE	256 (6.3%)
FLUTICASONE	2053 (4.4%)	CYCLOBENZAPRINE	248 (6.1%)
METOPROLOL	2035 (4.4%)	AZITHROMYCIN	198 (4.8%)
AMLODIPINE	2019 (4.3%)	METFORMIN	196 (4.8%)
NORETHINDRONE	1957 (4.2%)	ALBUTEROL	195 (4.8%)
SIMVASTATIN	1896 (4.1%)	METOPROLOL	190 (4.6%)
SERTRALINE	1887 (4.0%)	PREDNISONE	185 (4.5%)
AZITHROMYCIN	1629 (3.5%)	AMLODIPINE	182 (4.5%)
NORGESTIMATE	1366 (2.9%)	ZOLPIDEM	169 (4.1%)
BUPROPION	1306 (2.8%)	LOSARTAN	158 (3.9%)
ESCITALOPRAM	1280 (2.7%)	MELOXICAM	157 (3.8%)
CLAVULANIC ACID	1257 (2.7%)	PANTOPRAZOLE	150 (3.7%)
FLUOXETINE	1178 (2.5%)	ALPRAZOLAM	148 (3.6%)
MONTELUKAST	1146 (2.5%)	SERTRALINE	143 (3.5%)
PANTOPRAZOLE	1131 (2.4%)	FLUTICASONE	138 (3.4%)
DEXTROAMPHETAMINE	1112 (2.4%)	SIMVASTATIN	138 (3.4%)

*excluding opioid medications.

Table S3. The top 25 concomitant medications in patients receiving CYP2D6 opioids without and with CYP2D6 interacting co-medications.

**CYP2D6-activated Opioid_No interaction**		**CYP2D6-activated Opioid_With CYP2D6 interacting drugs**	
Medication Name	Number of patients (%)	Medication Name	Number of patients (%)
ACETAMINOPHEN	2094 (63.5%)	ACETAMINOPHEN	353 (61.2%)
AMOXICILLIN	326 (9.9%)	METOPROLOL	181 (31.4%)
GUAIFENESIN	285 (8.6%)	DULOXETINE	124 (21.5%)
IBUPROFEN	258 (7.8%)	BUPROPION	108 (18.7%)
HYDROCHLOROTHIAZIDE	252 (7.6%)	GABAPENTIN	104 (18.0%)
LISINOPRIL	235 (7.1%)	ATORVASTATIN	103 (17.9%)
GABAPENTIN	204 (6.2%)	LISINOPRIL	101 (17.5%)
CYCLOBENZAPRINE	190 (5.8%)	HYDROCHLOROTHIAZIDE	95 (16.5%)
ATORVASTATIN	188 (5.7%)	FLUOXETINE	89 (15.4%)
AZITHROMYCIN	180 (5.5%)	OMEPRAZOLE	79 (13.7%)
THYROXINE	175 (5.3%)	THYROXINE	67 (11.6%)
OMEPRAZOLE	168 (5.1%)	AMLODIPINE	63 (10.9%)
ALBUTEROL	159 (4.8%)	METFORMIN	61 (10.6%)
PREDNISONE	148 (4.5%)	PANTOPRAZOLE	58 (10.1%)
METFORMIN	123 (3.7%)	CYCLOBENZAPRINE	49 (8.5%)
MELOXICAM	122 (3.7%)	SIMVASTATIN	44 (7.6%)
ZOLPIDEM	120 (3.6%)	LOSARTAN	44 (7.6%)
ONDANSETRON	116 (3.5%)	FLUTICASONE	38 (6.6%)
PROMETHAZINE	110 (3.3%)	ZOLPIDEM	37 (6.4%)
AMLODIPINE	109 (3.3%)	PAROXETINE	36 (6.2%)
ETHINYLESTRADIOL	109 (3.3%)	CARVEDILOL	36 (6.2%)
LOSARTAN	109 (3.3%)	AMOXICILLIN	34 (5.9%)
SERTRALINE	104 (3.2%)	PREDNISONE	34 (5.9%)
CLAVULANIC ACID	100 (3.0%)	TRAZODONE	33 (5.7%)
ALPRAZOLAM	98 (3.0%)	MORPHINE	33 (5.7%)
